# Ensemble of ERDTs for Spectral–Spatial Classification of Hyperspectral Images Using MRS Object-Guided Morphological Profiles

**DOI:** 10.3390/jimaging6110114

**Published:** 2020-10-26

**Authors:** Alim Samat, Erzhu Li, Sicong Liu, Zelang Miao, Wei Wang

**Affiliations:** 1 State Key Laboratory of Desert and Oasis Ecology, Xinjiang Institute of Ecology and Geography, Chinese Academy of Sciences, Urumqi 830011, China; wangwei177@mails.ucas.ac.cn; 2 Research Center for Ecology and Environment of Central Asia, CAS, Urumqi 830011, China; 3 University of Chinese Academy of Sciences, Beijing 100049, China; 4 Department of Geographical Information Science, Jiangsu Normal University, Xuzhou 221100, China; liezrs2018@jsnu.edu.cn; 5 College of Surveying and Geoinformatics, Tongji University, Shanghai 200092, China; sicong.liu@tongji.edu.cn; 6 School of Geosciences & Info-Physics, Central South University, Changsha 410012, China; zelang.miao@csu.edu.cn

**Keywords:** MPs, OMPs, ERDT ensemble of ERDTs (EERDTs), ExtraTrees, multiresolution segmentation (MRS), hyperspectral, spectral-spatial classification

## Abstract

In spectral-spatial classification of hyperspectral image tasks, the performance of conventional morphological profiles (MPs) that use a sequence of structural elements (SEs) with predefined sizes and shapes could be limited by mismatching all the sizes and shapes of real-world objects in an image. To overcome such limitation, this paper proposes the use of object-guided morphological profiles (OMPs) by adopting multiresolution segmentation (MRS)-based objects as SEs for morphological closing and opening by geodesic reconstruction. Additionally, the ExtraTrees, bagging, adaptive boosting (AdaBoost), and MultiBoost ensemble versions of the extremely randomized decision trees (ERDTs) are introduced and comparatively investigated for spectral-spatial classification of hyperspectral images. Two hyperspectral benchmark images are used to validate the proposed approaches in terms of classification accuracy. The experimental results confirm the effectiveness of the proposed spatial feature extractors and ensemble classifiers.

## 1. Introduction

Due to the technical evolution of optical remote sensors over the last few decades, now the remote sensing (RS) community can obtain diverse data sets with rich spatial, spectral and temporal information. In particular, hyperspectral sensors can provide detailed spectral information with hundreds of spectral wavelengths and can increase the possibility of more accurately discriminating materials of interest. Furthermore, the high (5.0 m ≤ spatial resolution ≤ 10.0 m) and very high (spatial resolution < 5.0 m) spatial resolution (HR, VHR) of some of these sensors enables the analysis of small spatial structures with unprecedented detail. However, the high dimensionality of hyperspectral images may lead to the Hughes phenomenon, in which the classification accuracy will be downgraded in case of the limited number of training samples and the classification method is not capable of handling high-dimensional data [1]. Additionally, while HR and VHR data solve the problem of being able to “see” structure objects and elements, they do not help in focusing on the extraction procedure [2]. Therefore, solutions of spectral dimensionality reduction via feature selection (FS) and feature extraction (FE), and design of specific spectral-spatial classifiers have been identified for hyperspectral image classification in recent years [3,4]. 

In contrast with the works of using supervised, semi-supervised and unsupervised FS and FE methods for hyperspectral dimensionality reduction, the design of specific spectral-spatial classifiers has gained more interest from the fields of HR/VHR hyperspectral image processing. Especially when considering improvements on classification accuracy brought by spatial features. Hence, spatial contextual information has been widely incorporated into advanced machine learning (ML)-based classifiers such as support vector machines (SVMs) [5], extreme learning machines (ELMs) [6], ensemble learning methods such as AdaBoost [7], random forests (RaF) and rotation forests (RoF) [8,9], ExtraTrees [10], XGBoost [11], and deep neural networks (DNNs) [12], for multispectral, hyperspectral and full polarimetric synthetic aperture radar (PolSAR) image classification. 

According to their underlying model, spatial contextual information extraction approaches can be categorized into: (1) structural filter-based spatial processing methods using a fixed or adaptive structural element, such as edge-preserving filtering [13], local harmonic analysis [14,15,16], adaptive multidimensional Wiener filtering [17,18] and superpixel based filtering [19]; (2) random field models using a crisp neighborhood system, such as Markov random fields (MRF) [20,21], conditional random fields (CRF) [22] and discriminative random fields (DRF) [23]; (3) mathematical morphology (MM)-based approaches, such as morphological profiles (MPs) and extended MPs (EMPs) [24], object based MPs (OMPs) [25], attribute profiles (APs) [26], MPs with partial reconstruction (MPPR) [27], maximally stable extreme region guided MPs (MSER_MPs) [10] and its extended version EMSER_MPs [11]; (4) those based on image segmentation techniques [21,28,29]; (5) sparse representation based classification [30,31]; and (6) deep learning (DL)-based approaches [11,32,33,34]. Among these, MM-based approaches are likely the most widely used methods in the last ten years in the context of HR/VHR hyperspectral image processing [3,10,11,27].

Indeed, the advantages of MPs, EMPs, APs and MPPR in extracting spatial information from HR/VHR imagery has been clearly reported in many studies. However, being connected filters, they have the following limitations: (1) structural elements (SEs) with user-specified shape and size are inefficient for objects with diverse characteristics such as size, shape and homogeneity; (2) attribute filters (AFs) still suffer from the problem of leakage; and (3) limited numbers of SEs with specified sizes and shapes are unable to perfectly match all the sizes and shapes of the objects in a given image [3,10,27,35]. 

Inspired by the works in [25] and our previous work of [35], we adopt multiresolution segmentation (MRS)-based objects [36] as SEs for MPs extraction as an alternative solution. In particular, the original image is first segmented with the MRS technique as we demonstrated in [35]; in this procedure, multiscale values are provided for the purpose of multiscale spatial information extraction. Then, multiscale objects are exploited to extract OMPs by means of the basic principle of MM. Moreover, to avoid possible side effects from unusual minimum or maximum pixel values within objects, the OMPsM approach that contains additional mean pixel values within regions is proposed. Additionally, extended OMPs (EOMPs) and extended OMPsM (EOMPsM) are proposed by applying OMPs on the first three components after a principal component analysis (PCA) transformation executed on the spectral data. In contrast with OMPs in [25], we adopt MRS instead of arbitrary segmentation algorithm (ASA) for the image segmentation process, and shape and context features of objects are not considered in our MRS object-guided MPs. In contrast with our previous work in [35], object profiles such as roundness, compactness, rectangularity, density, asymmetry, border index, shape index, elliptic fit, minimum, maximum and standard deviation vales are not considered as well. At last, both works in [35] and [25] were conducted on multispectral imageries.

Following the quality of input features and a big enough set of training samples, the classifier robustness is the third component that affects the classification performance [8,37]. The extremely randomized decision tree (ERDT), a new tree induction algorithm that selects both attribute and cut-point splits, either totally or partially performed at random, and the ExtraTrees ensemble version were proposed for use in both classification and regression problems [38]. In our previous work, ERDT and ExtraTrees were investigated for their ability to classify three VHR multispectral images acquired over urban areas, and compared against decision tree (DT, C4.5), bagging, RaF, SVM and RoF in terms of classification accuracy and computational efficiency [10]. However, the performance of other ensemble versions (e.g., bagging, AdaBoost and MultiBoost) of ERDT for RS, particularly for hyperspectral image classification tasks using OMP and OMPsM features, has not yet been investigated. Hence, another contribution of this letter is to introduce and investigate the performance of bagging, AdaBoost and MultiBoost versions of ERDT in a hyperspectral image classification task.

## 2. Methods

### 2.1. Object-Guided MPs

Generally, morphological operators act on the values of the pixels by considering the neighborhood of the pixels determined by an SE with a predefined size and shape, based on two basic operators: dilation and erosion. In grayscale morphological reconstruction, two images and one SE are involved. One image, the marker *f*, contains the starting points for the transformation, while the other image, the mask *g*, constrains the transformation. According to the definitions from MM, morphological opening by reconstruction (OBR) of grayscale images can be obtained by first eroding (returning the minimum values of *f* contained in the specified SE) the input image and using it as a marker, while closing by reconstruction (CBR) can be obtained by complementing the marker image *f*, obtaining the OBR, and complementing the subsequent procedure [10,11,24,34,35]. In general, the object-guided morphological OBR can be obtained by first eroding the input image using segmented objects (where $J_{S}^{\lambda }$ represent the numbers (S) of objects from MRS procedure with scale λ) in the SE approach and by using the result as a marker in geodesic reconstruction by a dilation phase [35]:

(1) $$O_{R}^{Obj}(f)=R_{f}^{D}\left[\left(f⊙(\exists J_{i\in S}^{\lambda }\in J_{S}^{\lambda })\right)\right]$$

Similarly, we have

(2) $$C_{R}^{Obj}(f)=R_{f}^{E}\left[\left(f⊕(\exists J_{i\in S}^{\lambda }\in J_{S}^{\lambda })\right)\right]$$

where the object-guided CBR, obtained by complementing the image, contains the object-guided OBR (OOBR) using $\exists J_{i*}^{\prime }\in J_{*}^{\prime }$ as SEs and complements the resulting procedure:

(3) $$C_{R}^{Obj}(f)=R_{f}^{D\mathbb{C}}\left[\left(f^{\mathbb{C}}⊙(\exists J_{i\in S}^{\lambda }\in J_{S}^{\lambda })\right)\right]$$

In MM, the erosion of f by b at any location (x, y) is defined as the minimum value of all the pixels in its neighborhood defined by *b* ($\exists J_{i\in S}^{\lambda }\in J_{S}^{\lambda }$ in our case). In contrast, dilation returns the maximum value of the image in the window outlined by *b*. Then, the erosion and dilation operators can be defined as follows:

(4) $$\begin{array}{l} \left[f⊙(\exists J_{i\in S}^{\lambda }\in J_{S}^{\lambda })\right](x,y)=\underset{(s,t)\in J_{S}^{\lambda }}{\min}\left\{f(x+s,y+t)\right\}\\ \left[f⊕(\exists J_{i\in S}^{\lambda }\in J_{S}^{\lambda })\right](x,y)=\underset{(s,t)\in J_{S}^{\lambda }}{\max}\left\{f(x+s,y+t)\right\} \end{array}$$

Finally, if the structuring elements $\exists J_{i\in S}^{\lambda }\in J_{S}^{\lambda }$ are specified by objects, the OMPs of an image *f* can be defined as:

(5) $$OMPs(f)=\left[O_{R}^{Obj}(f),f,C_{R}^{Obj}(f)\right]$$

To avoid possible side effects from unusual minimum or maximum pixel values within objects, OMPsM are proposed by using extra mean pixel values that are contained within regions in an object-oriented manner:

(6) $$OMPsM(f)=\left[OMPs(f),OO_{mean}(f)\right]$$

Although the use of MPs can help in creating an image feature set that has more discriminative information, the redundancy is still evident in the feature set, particularly for hyperspectral images. Therefore, feature extraction can be used to find the most important features first; then, morphological operators are applied [24]. After PCA is applied to the original feature set, EOMPs and EOMPsM can be obtained by applying the basic principles of OMPs and OMPsM described above to the first few (typically three) features.

### 2.2. ExtraTrees

The ERDT approach is a new decision tree (DT) induction algorithm that selects attribute and cut-point splits, either completely or partially at random, whereas the ExtraTrees algorithm is an ensemble version of unpruned ERDT, which follows by introducing the random committee-based ensemble criterion [38]. By comparing ExtraTrees with other DT-based ensemble methods such as bagging, boosting and RaF, the main differences can be outlined: (1) this new algorithm splits nodes by choosing cut points (which are responsible for a significant part of the error rates of tree-based methods) fully at random in the tree induction phase, which makes the tree structures independent of the target variable values of the learning samples, and (2) it uses the entire set of learning samples, rather than a bootstrap replica sample (typically adopted by the other DT methods), to grow trees. 

Let $X={\left\{x_{\tau }\right\}}_{\tau =1}^{l}$ denote a labeled training set with $Y={\left\{y_{\tau }\right\}}_{\tau =1}^{l}$ as the labels, *K* represents the number of attributes randomly selected at each node, and η is the minimum sample size for splitting a node. An ERDT can be built by following the steps described in Algorithm 1.
**Algorithm 1** Algorithmic steps to build an extremely randomized decision tree (ERDT) [38].Inputs: labeled training set X, *K* and η.Build_ERDT(X, Y, *K*, η) as follows:
(1)Return a leaf labeled by class frequencies in X if (1) $\left|X_{l}\right|<\eta$ or (2) all candidate attributes are constant in X, or (3) the output variable is constant in X.(2)Otherwise:
Randomly select *K* attributes $\left\{a_{1},\ldots ,a_{K}\right\}$ without replacement among all candidates attributes;Generate *K* splits $\left\{s_{1},\ldots ,s_{K}\right\}|s_{i}=[a<a_{c}],\forall i=1,\ldots ,K$, where *a* is numerical attribute and $a_{c}$ is a cut-point uniformly drawn from $\left[a_{\min}^{X},a_{\max}^{X}\right]$, which denote the minimal and maximal values of a in X, respectively;Select a split $s^{*}=\underset{i=1,\ldots ,K}{\max}\left\{s_{i},X\right\}$;Split X into subsets $X_{l}$ and $X_{r}$ according to $s^{*}$;Build single ERDT $t_{f}^{ERDT}$ and $t_{r}^{ERDT}$ from subsets $X_{l}$ and $X_{r}$, respectively;Create a node with the split $s^{*}$, and attach $t_{f}^{ERDT}$ and $t_{r}^{ERDT}$ as left and right subtrees of this node;
Output: return the final resulting $t^{ERDT}$.


Thereafter, the ExtraTrees ensemble algorithm can be built exploiting the random committee ensemble learning (EL) criterion, i.e., an ensemble of randomizable base classifiers is built using a different random number seed, and the final prediction is the average of the predictions generated by the individual base classifiers [10,38]. Similarly, bagging, AdaBoost and MultiBoost versions of ERDT can be realized following the corresponding ensemble construction criteria. Bagging, also called bootstrap aggregating, trains each model in the ensemble using a randomly drawn subset of the training set, and then votes with equal weight [39]. AdaBoost, an abbreviation for adaptive boosting, incrementally builds an ensemble in the sense that subsequent weak learners are tweaked in favor of those instances misclassified by previous classifiers [40]. MultiBoost can be viewed as a combination of AdaBoost with bagging, which can harness both AdaBoost’s bias and variance reduction with bagging’s superior variance reduction to produce a committee with lower error, also offering, as an advantage over AdaBoost, the suitability to parallel execution [41].

## 3. Data Sets

**Figure 1:**
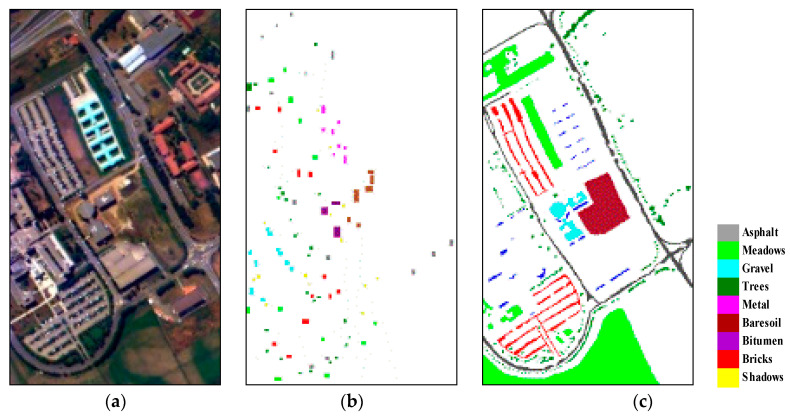
Pavia University data set: (**a**) color composite of the scene; (**b**) training set; (**c**) test set.

The first hyperspectral image was acquired by the Reflective Optics System Imaging Spectrometer (ROSIS) optical sensor, which provides 115 bands with a spectral range coverage ranging from 0.43 μm to 0.86 μm. The main objective of the ROSIS project is the detection of spectral fine structures especially in coastal waters. This task determined the selection of the spectral range, bandwidth, number of channels, radiometric resolution and its tilt capability for sun glint avoidance. However, ROSIS can be used just as well for the monitoring of spectral features above land or within the atmosphere. The image shown in Figure 1a depicts the Engineering School of Pavia University (Pavia, Italy) with the geometric resolution of 1.3 m. The image has 610 × 340 pixels with 103 spectral channels, where 12 very noisy bands were discarded manually after the data acquisition. The validation data refer to nine land cover classes (as shown in Figure 1). This scene was provided by Professor Paolo Gamba from the Telecommunications and Remote Sensing Laboratory, Pavia University (Pavia, Italy).

**Figure 2:**
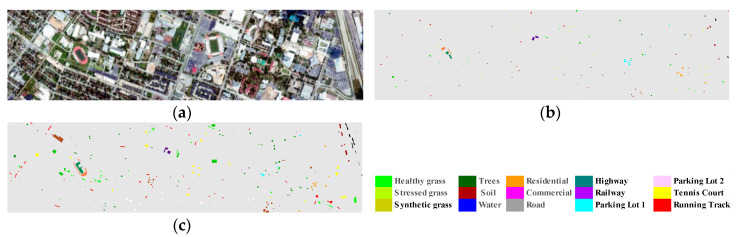
GRSS-DFC2013 data set: (**a**) color composite of the scene; (**b**) training set; (**c**) test set.

The second hyperspectral image was acquired at a spatial resolution of 2.5 m by the National Science Foundation (NSF) -funded Center for Airborne Laser Mapping (NCALM) over the University of Houston campus and the neighboring urban area, on 23 June 2012 (Figure 2). The 15 classes of interest selected by the Institute of Electrical and Electronics Engineers Geoscience and Remote Sensing Society (IEEE GRSS) Image Analysis and Data Fusion Technical Committee for organizing the 2013 Data Fusion Contest (DFC) are reported for both the training and validation sets [42]. Originally, this image has 349 × 1905 pixels with 144 spectral bands in the spectral range between 380 and 1050 nm. In our experiment, dense cloud-covered area at the right part and total of nine blank pixel lines at the upper and lower image edges were removed, which result in subset image with the size of 340 × 1350 pixels.

## 4. Results

### 4.1. Experimental Configuration

The free parameters of ERDT, where *K* represents the number of the attributes set, are the same as the default for the C4.5 algorithm used in bagging and RaF. The overall accuracy (OA) and kappa statistic are used to evaluate the classification performances of these methods. In the case of multiclass classification, OA is usually calculated by dividing the sum of diagonal numbers, which represent correctly classified instances, by the total number of reference instances in the confusion matrix:

(7) $$OA=\frac{\sum_{i}^{N}Tp_{i}}{\sum_{i}^{N}Tn_{i}}$$

where $Tp_{i}$ represents numbers of the correctly classified instances for class *i*, $Tn_{i}$ represents the total number of instances from class *i*, and there are a total of *N* classes.

To generate MPs and MPPR, we apply a disk shape SE with *n* = 10 openings and closings by conventional and partial reconstructions, ranging from one to ten with a step-size increment of one. This choice results in a total of 2163 = 103 + 103 × 10 × 2 and 3024 = 144 + 144 × 10 × 2 dimensional stacked data sets using original spectral bands and a total of 70 = 10 + 3 × 10 × 2 and 67 = 7 + 3 × 10 × 2 dimensional stacked data sets using PCA-transformed features, for Pavia University and GRSS-DFC2013, respectively. Note that only the first ten and seven PCA-transformed features from Pavia University and GRSS-DFC2013, respectively, are considered in the experiments. For fair comparison purposes, we set a total of ten scales for MRS in the image segmentation phase. For instance, the scale parameter is increased from 10 to 55 by a step-size of five to produce a total of 10 scale segmentation results. The segmentation result, which is crucial for guiding MPs, typically relies on the scale parameters that are highly dependent on the spatial resolution and geometrical complexity of the image under consideration. Hence, in the next experiment, we examine the performance of OMPs, OMPsM, EOMPs and EOMPsM with different scale sets. Note that OMPsM and EOMPsM also contain the mean pixel values within objects that produce 3193 = 103 + 103 × 10 × 3, 4464 = 144 + 144 × 10 × 3, 100 = 10 + 3 × 10 × 3 and 97 = 7 + 3 × 10 × 3 dimensional stacked data sets using the original spectral bands and PCA-transformed features for Pavia University and GRSS-DFC2013, respectively.

### 4.2. Results and Analysis

**Figure 3:**
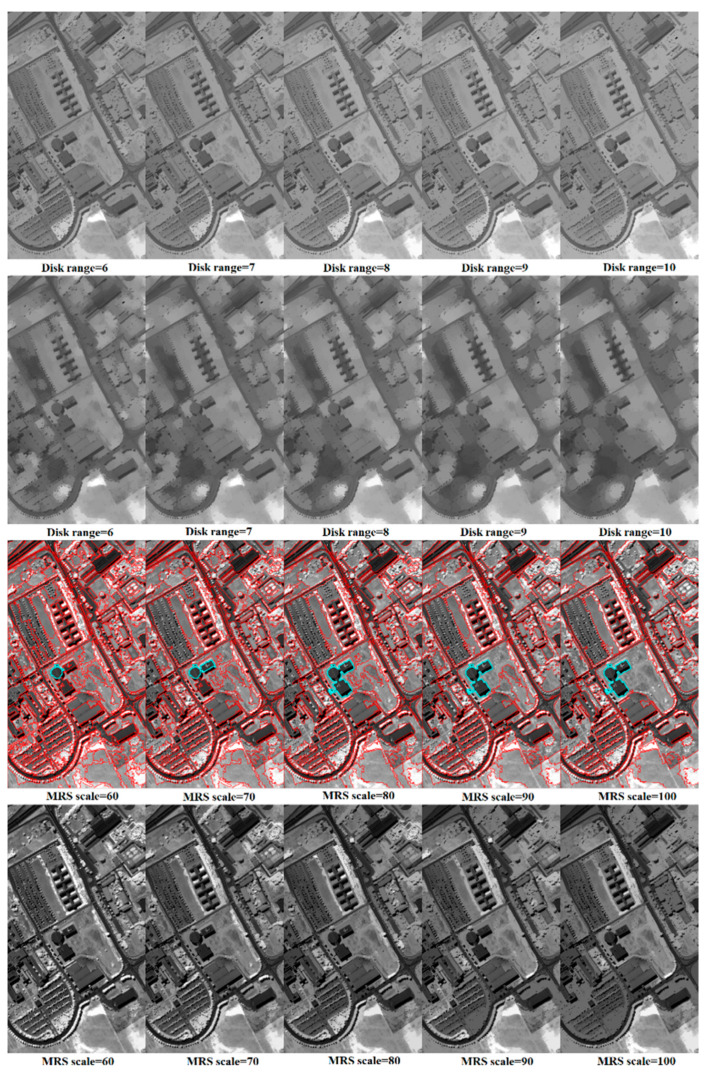
Examples of opening by reconstruction (OBR) (row 1), opening by partial reconstruction (OBPR) (row 2), multiresolution segmentation (MRS) objects (row 3) and object-guided opening by reconstruction (OOBR) (row 4) with different parameter sets from the second principal component of the Pavia University data.

Figure 3 shows the examples of OBR, opening by partial reconstruction (OBPR), and the proposed OOBR with different parameter sets using the second principal component of the Pavia University data. The range of disk shape SEs in OBR and OBPR were set between 6 to 10, while the scale parameters of MRS and OOBR were set between 60 to 100 empirically. A comparison of the results in the first row indicates that OBPR is more capable of modeling the attributes of different objects than OBR from a sequence of SEs, in accordance with the finding in [12]. However, many large objects and boundaries between different objects that should have appeared were removed at a very small scale after OBPR. In contrast, OOBR maintains the object information between boundaries exactly as in the original by affecting only the brightness or darkness of the objects with different scale parameters. In other words, effects from the scale parameter of OOBR are much smaller than effects from the scale of OBPR.

**Figure 4:**
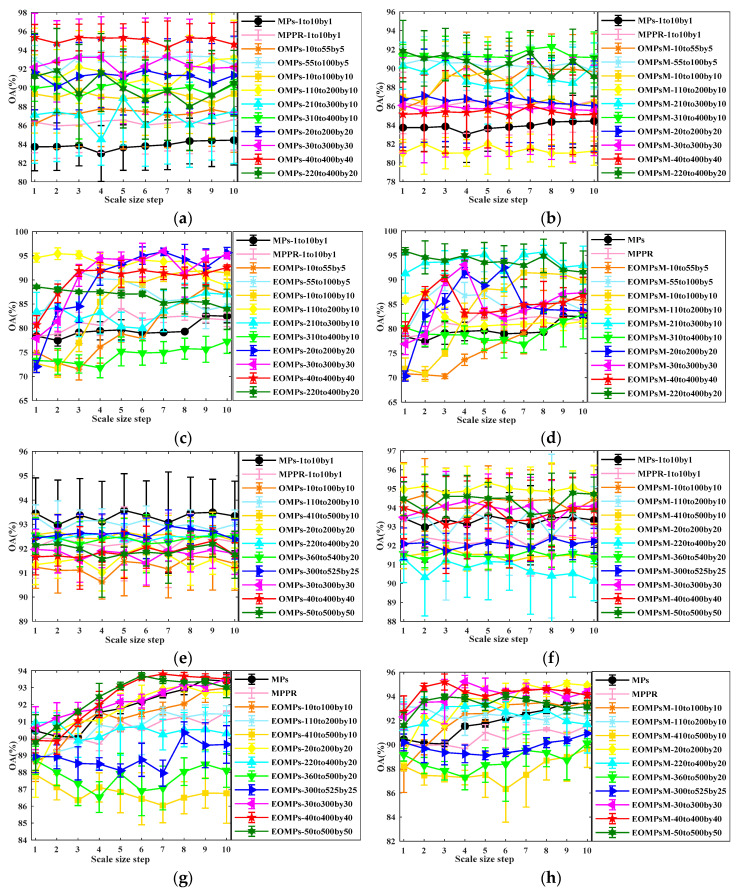
Mean overall accuracy (OA) values versus different sizes of structural elements (SEs) and segmentation scale sets in morphological profiles (MPs), morphological profiles with partial reconstruction (MPPR), extended object-guided morphological profiles (EOMPs) and EOMPs with mean values (EOMPsM) on raw spectral (**a**,**b**,**e**,**f**) and principal component analysis (PCA)-transformed (**c**,**d**,**g**,**h**) features from Reflective Optics System Imaging Spectrometer (ROSIS) university (row 1) and GRSS-DFC2013 (row 2) data sets using an support vector machine (SVM).

In Figure 4, we present the results for various spatial feature extractors with different parameter sets using an SVM with an radial bias function (RBF) kernel to evaluate the performance of EOMPs and EOMPsM on the considered data sets. A total of 10 rounds were executed for each experiment for the purpose of an objective evaluation.

The graphs confirm the superiority of the proposed feature extraction methods in contrast to MPs and MPPR, specifically with the best improvements obtained by EOMPsM, and this is valid for both data sets (Figure 4c,d,g,h). However, the effects of the segmentation scale parameter in MRS are different for the considered data sets using the original spectral and the PCA-transferred features. For instance, the best OA curves are achieved by OMPs using the original spectral bands of ROSIS university data with the MRS scale ranges set as 40 to 400 with 40 sequence steps (see Figure 4a). In contrast, the best OA curves are achieved by OMPsM using the original spectral bands with the MRS scale range set from 310 to 400, with 10 sequence steps (see Figure 4b). 

Interestingly, the superiority of the larger scale set relative to the smaller one is no longer true when using the original spectral features, because some noise corrupted bands mislead partial reconstruction in MPs and MPPR and the image segmentation procedures in OMPs and OMPsM (see Figure 4a,b,e,f). Additionally, EOMPsM with a larger scale set could limit and even degrade the classification accuracy, whereas a single mean value was assigned to different targets contained in single large segmented objects (see Figure 4c,d,g,h). Summarizing these results, OMPs and EOMPs are more suitable to accommodate the original spectral and PCA-transformed features for a larger MRS scale range set with a larger sequence step, while OMPsM and EOMPsM are more suitable for a larger MRS scale range but with a smaller sequence step.

**Figure 5:**
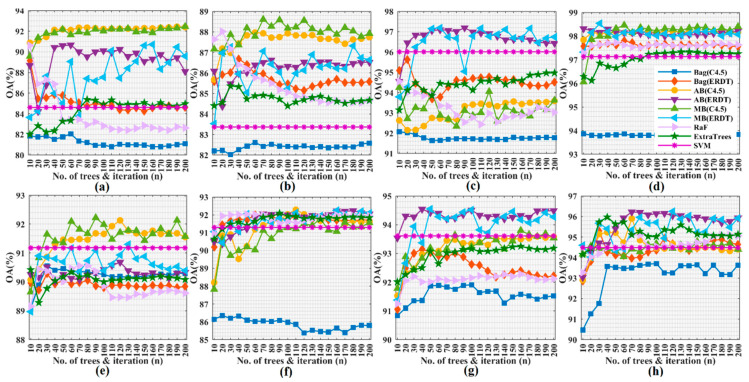
OA values versus the number of trees/iterations in bagging (Bag(C4.5), Bag(ERDT)), AdaBoost (AB(C4.5), AB(ERDT)), MultiBoost (MB(C4.5), MB(ERDT)), RaF(C4.5) and ExtraTrees using MPs (**a**,**e**), MPPR (**b**,**f**), object-guided morphological profiles (OMPs) (**c**,**g**) and OMPsM (**d**,**h**) from PCA-transformed features of Pavia University (**a**–**d**) and GRSS-DFC2013 (**e**–**h**) data sets.

Figure 5 shows the OA values with respect to the number of trees in bagging, RaF and ExtraTrees, and with respect to the number of iterations in AdaBoost and MultiBoostAB ensemble classifiers. According to these graphs, there are no prominent improvements or decreasing trends for a tree size greater than 100 in most of the cases, a result consistent with the findings in other studies [8,10]. Moreover, it is clear that the bagging ensemble of ERDT (Bag(ERDT)) is uniformly better than the bagging ensemble of the conventional C4.5 approach (Bag(C4.5)) in all classification scenarios in terms of classification accuracy. Instead, the performance of the MultiBoostAB and AdaBoost ensemble of ERDT (MB(ERDT) and AB(ERDT)) are not constantly superior to the MultiBoostAB and AdaBoost ensemble of C4.5 (MB(C4.5) and (C4.5)) using different features on the two data sets. Specifically, MB(C4.5) and AB(C4.5) show better OA values than MB(ERDT) and (ERDT) using MPs and MPPR but show lower OA values using OMPs features for considered data sets, and similar OA values shown by using OMPsM features of Pavia University data set but lower values by using OMPsM features from the GRSS-DFC2013 data set. MB(ERDT) and AB(ERDT) show better results using OMPs and OMPsM features but lower results using MPs and MPPR features, which can be explained by the fact that: (1) fewer, but harder to be correctly classified, instances are those focused on by the MultiBoostAB and AdaBoost criteria, which could further weaken ERDT and lead to an overly abundant diversity that hindered the construction of an improved ensemble scenario, (2) however, this shortage could be overcome by exploiting advanced discriminative features. Other solutions for this limitation could be either (1) early stopping of the ensemble or (2) critical tuning of the parameters of ERDT in each iteration step.

**Figure 6:**
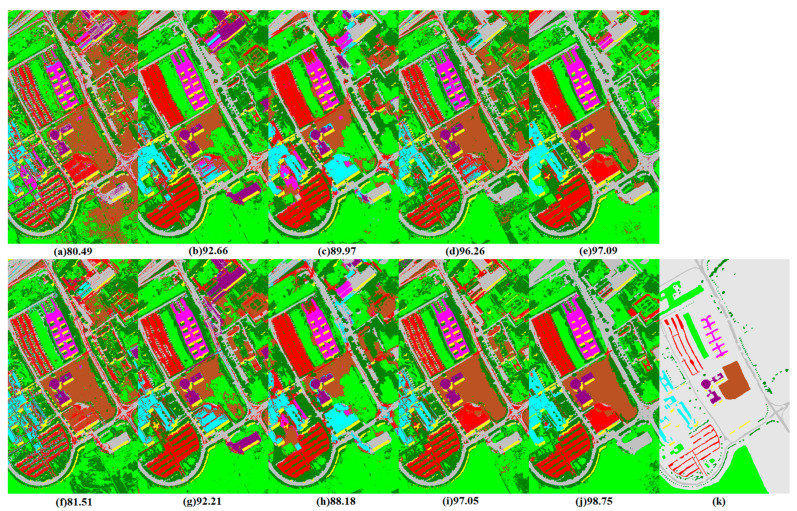
Classification maps with OA values (**a**–**j**) corresponding to Table 1 and ground truth map, (**k**) for comparison with Pavia University test data.

**Figure 7:**
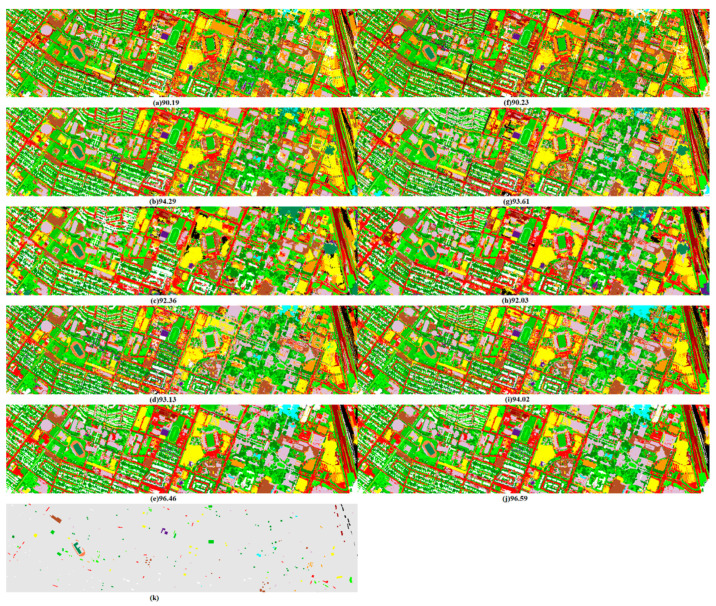
Classification maps with OA values (**a**–**j**) corresponding to Table 2 and ground truth map, (**k**) for comparison with GRSS-DFC2013 test data.

Finally, Figure 6 and Figure 7 present the best classification maps corresponding to the highlighted values in Table 1 and Table 2, with OA and the kappa statistics for MPs, MPPR, OMPs and OMPsM features extracted from the original raw bands or the PCA-transformed features. 

Once again, all classifiers uniformly and clearly confirm the effectiveness of the proposed MRS OMPs over the conventional MPs and MPPR approaches. For instance, the best classification results, with the highest OA (98.75%) and kappa statistic (0.98) values, were achieved by MultiBoost(C4.5) using EOMPsM features on the ROSIS university data, and by AdaBoost(ERDT) using OMPsM features on GRSS-DFC2013 data (OA = 96.59%, kappa statistic = 0.96). If we compare the ensemble versions of ERDT, it is clear that ExtraTrees is better than Bag(C4.5) and is comparable to RaF(C4.5), and that the best improvement in OA values is achieved by either AdaBoost or MultiBoost ensemble (see the numbers in bold in Table 1 and Table 2). Additionally, regarding EOMPs, the superiority of EOMPsM over EMPs is clear, its performance is comparable to the one by EMPPR, or even better in some cases.

## 5. Conclusions

In this study, we propose the concept of OMPs for spatial feature extraction in high-resolution hyperspectral images, by using multiscale objects after multi resolution segmentation as the SEs. Additionally, ExtraTrees, bagging, AdaBoost, and MultiBoost ensemble versions of the ERDT algorithm are introduced and comparatively investigated on two benchmark hyperspectral data sets. The experimental results confirm the effectiveness of the proposed OMPs, OMPs(M) and their extended versions. In addition, the superiority of EOMPsM over the conventional MPs and MPPR is reported. In the evaluation of the adopted classifiers, the bagging ensemble of ERDT is better than the bagging version of C4.5, and ExtraTrees is better than Bag(C4.5) but comparable to RaF(C4.5). The best improvements are reached by the AdaBoost or MultiBoost ensemble of ERDT using OMPsM extracted from the original bands, or EOMPsM extracted from the PCA-transformed features.

Future works will focus on the role of self-adaptive segmentation scale selection for multiscale segmentation in the usefulness of OMPs and EOMPsM. The early steps and self-adaptive parameter tuning of individual ERDT in the AdaBoost and MultiBoost ensemble framework will also be investigated.

## Figures and Tables

**Table 1:** The best OA and kappa statistics for considered classifiers using MPs, MPPR, OMPs and OMPsM features from PCA-transformed features of Pavia University data (F1: MPs, F2: MPPR; F3: OMPs; F4: OMPsM).

Ensemble Method	Classifier	Raw:	+F1	+F2	+F3	+F4	PC10:	+F1	+F2	+F3	+F4
None	C4.5	65.76,0.58	77.42,0.71	82.60,0.77	83.51,0.80	91.89,0.89	73.17,0.66	78.45,0.73	80.34,0.74	92.05,0.90	95.84,0.95
	ERDT	64.66,0.57	79.61,0.74	81.72,0.76	83.62,0.79	91.74,0.89	62.42,0.54	71.83,0.65	73.39,0.66	90.15,0.87	95.06,0.94
	SVM	**80.49,0.76**	87.31,0.84	87.24,0.83	**96.26,0.95**	92.24,0.90	**81.51,0.77**	84.61,0.80	83.79,0.79	91.49,0.89	91.13,0.89
Bagging	C4.5	72.18,0.66	79.90,0.74	86.74,0.82	95.94,0.95	91.15,0.88	76.36,0.70	79.90,0.74	86.74,0.82	95.26,0.94	97.75,0.97
	ERDT	71.42,0.65	84.63,0.80	88.74,0.85	87.76,0.84	95.01,0.93	78.56,0.73	80.97,0.76	82.42,0.77	95.95,0.95	98.43,0.98
AdaBoost	C4.5	74.18,0.68	92.44,0.90	88.94,0.86	93.80,0.92	91.41,0.89	76.99,0.71	**92.21,0.90**	87.93,0.84	94.48,0.93	98.66,0.98
	ERDT	73.67,0.68	88.84,0.84	89.35,0.86	91.15,0.88	**97.09,0.96**	77.40,0.72	88.42,0.85	86.28,0.82	**97.05,0.96**	98.29,0.98
MultiBoost	C4.5	73.91,0.68	**92.66,0.90**	88.80,0.85	94.89,0.93	90.52,0.87	77.49,0.72	92.01,0.89	**88.18,0.84**	94.52,0.93	**98.75,0.98**
	ERDT	72.79,0.66	88.15,0.84	**89.97,0.87**	90.56,0.87	94.22,0.92	77.63,0.72	83.96,0.79	84.92,0.80	96.71,0.96	98.51,0.98
Random Forest	C4.5	71.08,0.64	85.65,0.81	87.88,0.84	90.93,0.88	92.94,0.91	76.31,0.70	82.83,0.78	85.06,0.80	94.29,0.92	97.66,0.97
ExtraTrees	ERDT	72.70,0.66	86.58,0.82	84.09,0.79	88.54,0.85	94.96,0.93	76.09,0.70	85.23,0.81	84.60,0.80	95.24,0.94	97.80,0.96

**Table 2:** The best OA and kappa statistics for considered classifiers using MPs, MPPR, OMPs and OMPsM features from PCA-transformed features of GRSS-DFC2013 data (F1: MPs, F2: MPPR; F3: OMPs; F4: OMPsM).

Ensemble Method	Classifier	Raw:	+F1	+F2	+F3	+F4	PC10:	+F1	+F2	+F3	+F4
None	C4.5	80.07,0.78	88.93,0.88	88.48,0.87	89.76,0.89	91.43,0.91	83.51,0.82	86.54,0.85	83.97,0.83	88.64,0.88	91.59,0.91
	ERDT	78.74,0.77	88.35,0.87	85.27,0.84	89.07,0.88	92.39,0.92	81.40,0.80	86.98,0.86	83.90,0.82	88.16,0.87	89.41,0.88
	SVM	**90.19,0.89**	94.19,0.94	90.42,0.90	**93.13,0.93**	**96.46,0.96**	89.72,0.89	**93.61,0.93**	91.42,0.91	93.51,0.93	95.18,0.95
Bagging	C4.5	84.90,0.84	90.73,0.90	79.44,0.78	90.87,0.90	94.06,0.94	86.59,0.85	90.26,0.89	85.98,0.85	94.18,0.94	96.30,0.96
	ERDT	86.24,0.85	93.50,0.93	92.06,0.91	92.59,0.92	94.71,0.94	89.78,0.89	90.90,0.90	91.75,0.91	93.91,0.93	96.13,0.96
AdaBoost	C4.5	85.94,0.85	91.44,0.91	89.78,0.89	92.75,0.92	94.04,0.93	88.36,0.87	91.72,0.91	91.54,0.91	91.91,0.91	94.61,0.94
	ERDT	86.50,0.85	**94.29,0.94**	92.64,0.92	92.91,0.92	94.80,0.94	89.41,0.88	90.81,0.90	**92.03,0.91**	**94.42,0.94**	**96.59,0.96**
MultiBoost	C4.5	86.16,0.85	91.54,0.91	89.73,0.89	92.65,0.92	94.34,0.93	88.45,0.87	91.98,0.91	91.27,0.90	93.77,0.93	96.06,0.96
	ERDT	86.58,0.85	93.68,0.93	91.78,0.91	93.05,0.92	94.86,0.94	**90.23,0.89**	91.87,0.91	91.99,0.91	93.98,0.93	95.66,0.95
Random Forest	C4.5	85.25,0.84	93.61,0.93	91.30,0.91	92.93,0.92	94.65,0.94	88.95,0.88	89.86,0.89	91.63,0.91	92.15,0.91	95.76,0.95
ExtraTrees	ERDT	86.91,0.86	93.20,0.93	**92.36,0.92**	92.68,0.92	95.06,0.95	89.28,0.88	90.19,0.89	91.70,0.91	93.13,0.93	95.26,0.95
